# The INVEST project: investigating the use of evidence synthesis in the design and analysis of clinical trials

**DOI:** 10.1186/s13063-017-1955-y

**Published:** 2017-05-15

**Authors:** Gemma L. Clayton, Isabelle L. Smith, Julian P. T. Higgins, Borislava Mihaylova, Benjamin Thorpe, Robert Cicero, Kusal Lokuge, Julia R. Forman, Jayne F. Tierney, Ian R. White, Linda D. Sharples, Hayley E. Jones

**Affiliations:** 1 School of Social and Community Medicine, Faculty of Health Sciences, University of Bristol, Canynge Hall, 39 Whatley Road, Bristol, BS8 2PS UK; 2 Leeds Institute of Clinical Trials Research, University of Leeds, Leeds, UK; 3 Health Economics Research Centre, Nuffield Department of Population Health, University of Oxford, Oxford, UK; 4 Cambridge Clinical Trials Unit, University of Cambridge, Cambridge, UK; 5 MRC Clinical Trials Unit, University College London, London, UK; 6 MRC Biostatistics Unit, Cambridge Institute of Public Health, Cambridge, UK; 7 London School of Hygiene and Tropical Medicine, London, UK

**Keywords:** Systematic review, Meta-analysis, Network meta-analysis, Decision models, Value of information analysis, Sample size calculations, Informative prior distributions, Bayesian analysis

## Abstract

**Background:**

When designing and analysing clinical trials, using previous relevant information, perhaps in the form of evidence syntheses, can reduce research waste. We conducted the INVEST (INVestigating the use of Evidence Synthesis in the design and analysis of clinical Trials) survey to summarise the current use of evidence synthesis in trial design and analysis, to capture opinions of trialists and methodologists on such use, and to understand any barriers.

**Methods:**

Our sampling frame was all delegates attending the International Clinical Trials Methodology Conference in November 2015. Respondents were asked to indicate (1) their views on the use of evidence synthesis in trial design and analysis, (2) their own use during the past 10 years and (3) the three greatest barriers to use in practice.

**Results:**

Of approximately 638 attendees of the conference, 106 (17%) completed the survey, half of whom were statisticians. Support was generally high for using a description of previous evidence, a systematic review or a meta-analysis in trial design. Generally, respondents did not seem to be using evidence syntheses as often as they felt they should. For example, only 50% (42/84 relevant respondents) had used a meta-analysis to inform whether a trial is needed compared with 74% (62/84) indicating that this is desirable. Only 6% (5/81 relevant respondents) had used a value of information analysis to inform sample size calculations versus 22% (18/81) indicating support for this. Surprisingly large numbers of participants indicated support for, and previous use of, evidence syntheses in trial analysis. For example, 79% (79/100) of respondents indicated that external information about the treatment effect should be used to inform aspects of the analysis. The greatest perceived barrier to using evidence synthesis methods in trial design or analysis was time constraints, followed by a belief that the new trial was the first in the area.

**Conclusions:**

Evidence syntheses can be resource-intensive, but their use in informing the design, conduct and analysis of clinical trials is widely considered desirable. We advocate additional research, training and investment in resources dedicated to ways in which evidence syntheses can be undertaken more efficiently, offering the potential for cost savings in the long term.

**Electronic supplementary material:**

The online version of this article (doi:10.1186/s13063-017-1955-y) contains supplementary material, which is available to authorized users.

## Background

When designing and analysing a clinical trial, it is important to look at previous evidence and use relevant information to inform aspects of the new trial, thereby reducing waste in research [1]. Previous evidence should firstly be used to assess whether a gap in the current evidence base justifies a new trial [2, 3]. Subsequently there are many possible uses of previous evidence in informing the planning of a trial before it begins, monitoring of a trial in progress, and analysis and reporting of the results of a new trial alongside other relevant research [4, 5] (Table 1). In the design stage, existing evidence can be used to refine the choice of population, control treatment, intervention, definition of an outcome and duration of follow-up in order to maximise relevance of the findings [6, 7]. Previous studies might inform the choice of most appropriate statistical analysis (e.g. based on how rare the outcome is), while quantitative information on the likely treatment effect or the event rate in the control group might be used in sample size calculations [8, 9]. In the analysis stage, external information could be used to improve precision in estimation as part of a secondary analysis, particularly for parameters that are poorly estimated; for example, the intra-class correlation coefficient (ICC) in a cluster randomised trial [10, 11] or baseline event rates if events are rare. To aid interpretation of trial results in the context of relevant research [12], we might be interested in examining results from an updated meta-analysis [13, 14] or the results of a Bayesian analysis of the new trial in which an informative prior distribution for the intervention effect (based on results of earlier studies) has been incorporated. The analyst could also attempt to account for potential flaws in the methodology of the new trial, such as the allocated treatment being unblinded to the patient or personnel, which can cause bias in the treatment effect estimate [15]. External evidence about such bias might come from ‘meta-epidemiological’ studies and could be used to adjust the treatment effect estimate from the new study [16], allowing the analyst to assess the sensitivity of the findings.

**Table 1:** Summary of opportunities for evidence synthesis to inform design, conduct and analysis of a clinical trial

Stages of a clinical trial	Opportunities in which previous evidence might be used
Prior to design	To justify the need for a new trial in light of the existing evidence base. A systematic literature review and, where appropriate, quantitative synthesis, could be used to assess the need for the new trial [31, 32]. If there are no relevant previous trials, a search strategy might be requested by funders, such as the ﻿National Institute for Health Research (NIHR), to support this. Relevant systematic reviews might include existing clinical trials, early-phase trials, nonrandomised comparisons, animal studies or qualitative research studies [7].
Design	Choice of population. A systematic review may highlight the population and particular subgroups that warrant further investigation [17].
	Choice of interventions and comparators. Results from evidence syntheses, including network meta-analyses, decision models and value of information analyses, can be used to choose which interventions and comparators to trial [33] and characteristics of these, e.g. dose or duration of treatment [4, 17].
	Choice of outcomes and length of follow-up. A systematic review may help inform the choice of outcomes [4, 7] in a new trial and how they should be defined and, if relevant, the duration of follow-up [17]. For example, a systematic review may highlight adverse events that should be monitored, in particular events that are expected and related.
	Sample size calculations A systematic review and/or meta-analysis may provide information on the parameters needed for sample size calculations [4, 17] such as the standard deviation, control group outcome rates, plausible effect sizes, loss to follow-up and correlation coefficients [7]. Alternatively, expected value of sample information calculations can be used to assess the ability of a new trial to inform cost-effectiveness assessment of the intervention and reduce decision uncertainty [29].
	Recruitment and consent. For example, good or poor recruitment rates in previous relevant trials can inform site selection in a new multicentre trial [4].
Monitoring (conduct)	To deal with adverse events Observed adverse event rates can be compared with predictions from a synthesis of historic data to see if they are higher than expected by chance [34].
	To decide whether to stop an ongoing trial Emerging trial results considered in the context of results from previous studies might be used to make the decision to stop a trial early [17].
Analysis	To inform the statistical analysis plan Factors, such as measures of effects (event rates, mean difference, etc.) from previous trials, might influence the choice of statistical model. Prognostic or predictive factors identified though evidence synthesis may be used to stratify or adjust trial analyses [17]. Choice of the most important covariates to be recorded for imputation modelling might be informed by patterns of missing data in previous trials.
	To assess the trial treatment effect in the context of existing evidence An existing meta-analysis might be used to form a prior distribution for the treatment effect in a new study which can then be updated using the trial data in a Bayesian statistical analysis.
	To adjust for potential biases. External evidence about typical biases associated with undesirable study characteristics, e.g. inadequate blinding, might come from ‘meta-epidemiological’ studies [35], allowing the analyst to assess the sensitivity of the findings to alternative model assumptions.
	To inform secondary parameters. External evidence might be used to improve the estimation of ‘nuisance’ parameters involved in trial analysis which are often poorly estimated, such as the intra-class correlation coefficient (ICC) in cluster randomised trials [10] and between-centre variability in multicentre trials.
Reporting	To report the new trial results in the context of the wider evidence base. An updated systematic review [12] or meta-analysis including the new trial results [36] should be reported to put the results in the context of the wider evidence base [17].

In a survey of 24 investigators whose trials were included in an update of a Cochrane review, only 8 (33%) indicated that a previous review had influenced trial design and only 2 (8%) had used the previous Cochrane review [2, 3]. More recently, reviews of trials funded by the National Institute for Health Research (NIHR) Health Technology Assessment (HTA) programme found that the majority (77% of those funded between 2006 and 2008 [4] and 100% of those funded in 2013 [7]) referenced a systematic review in the funding application. When a systematic review was not referenced, there were valid reasons for this such as there being no relevant systematic review addressing the proposed research question [7]. Arguably of more interest is whether and how a cited review was *used* to inform trial design. The recent review of Bhurke et al. [7] found that 94% (32/34) of the trials examined used the referenced systematic review to justify the treatment comparison in the new trial, but that other uses were relatively infrequent. The other most common uses were in selection of a definition or outcome (16%), to inform the standard deviation (9%) or to inform duration of follow-up (6%). Tierney et al. describe examples of how meta-analyses of individual participant data (IPD) have informed trial design, conduct and analysis in practice [17]. To our knowledge, there are no recent studies investigating the extent of the use of evidence synthesis in the design of trials funded through streams other than the NIHR HTA programme or in trial analyses.

Here, we report results from the INVEST (INVestigating the use of Evidence Synthesis in the design of clinical Trials) survey. The main objectives of the survey were to summarise the current use of evidence synthesis in trial design and analysis across clinical trials teams, to capture current opinions of trialists and methodologists on such use, and to understand any barriers to use in practice.

## Methods

The sampling frame consisted of all delegates at the 2-day International Clinical Trials Methodology Conference (ICTMC) on 16–17 November 2015. The conference was open to both those involved and those who have an interest in clinical trials methodology. Approximately 638 people registered to attend the conference across a range of disciplines including trialists, clinicians, statisticians, health economists, information specialists and qualitative researchers. Ninety-five percent of the registered delegates were from the UK and the Republic of Ireland, with the remaining 5% from Australia, Canada, Denmark, France, Germany, Holland and the United States. The main UK research centres represented were Aberdeen, Birmingham, Bristol, Cambridge, Cardiff, Coventry, Glasgow, Leeds, London, Liverpool, Manchester, Oxford and Southampton. Conference delegates were first invited to take part in the survey during the opening plenary session, then by researchers from the INVEST team during breaks. The survey could be completed either on paper or online, with a closing date of 18 December 2015. The survey in full is available in Additional file 1.

Following details about their job role, job setting and the length of time that they had spent working in clinical trials, respondents who indicated that they had been involved in trial design (and/or analysis) were further asked questions about whether, and how, they have used evidence synthesis in practice. All respondents were then asked about their views on the use of evidence synthesis in trial design and analysis. They were also asked to rank what they considered to be the three greatest barriers to such use. There were nine potential barriers listed including an ‘other’ category allowing free text. The subsets of respondents who indicated that they had been involved in trial design (and/or analysis) were used to contrast views on whether evidence synthesis methods *should* be used versus current use in practice.

### The use of evidence synthesis to inform trial design

Respondents who indicated they had personally been involved in trial design were asked to consider any trials in which they had been involved over the last 10 years and to specify, if applicable, how evidence synthesis had been used in practice. A matrix style layout was chosen to allow multiple responses, with rows for each area of trial design and columns for types of evidence synthesis. In addition to (1) a description of previous evidence, (2) a systematic review and (3) a meta-analysis, we listed three evidence synthesis methods that extend meta-analysis: (4) network meta-analysis (NMA) which allows the simultaneous comparison of the effectiveness of multiple interventions through the use of direct and indirect evidence, (5) an economic decision model which can be used to evaluate intervention effects formally in the context of other factors, such as costs and potential harms, and make decisions on the use of interventions in practice, (6) a value of information (VoI) analysis which is sometimes used to assess whether there is value in conducting a new study, and to identify the optimal design for such a study within an analytical modelling framework [18]. A final option of ‘none of these methods’ was included. Respondents were provided with a brief definition of these evidence synthesis methods to reduce ambiguity. The areas of trial design listed were: (1) whether a trial is needed, (2) the choice of population, (3) the choice of interventions, (4) the choice of outcomes and follow-up time and (5) sample size calculations. Respondents were also asked to indicate whether any evidence synthesis used had been performed by the trial team or previously published by others.

We also asked all respondents which of the listed evidence synthesis methods they thought *should* be used to inform aspects of trial design. This question was formatted to match the earlier question about how those involved in trial design were using evidence synthesis methods, facilitating comparison between ideal and current practices.

### The use of evidence synthesis to inform trial analysis

Respondents who indicated they had personally been involved in trial analysis were asked which (if any) of three types of external evidence they had used in practice, during the last 10 years: (1) external information about the treatment effect (including a meta-analysis), (2) evidence around the likely size of potential biases arising from trial conduct (e.g. blinding infeasible) and (3) other quantities involved in the analysis (e.g. correlations or baseline event rates).

We asked all survey respondents whether each of these three types of external evidence *should* be used to inform trial analysis. For each of these, the options were ‘yes’, ‘no’ and ‘don’t know’. An overall ‘don’t understand’ response was also included since we anticipated that some of these uses of evidence synthesis might be new concepts to some respondents.

### Analysis of survey responses

Our analysis is descriptive, as sample sizes were not sufficient for a robust assessment of associations or subgroup comparisons. Missing responses were excluded from denominators and are indicated in footnotes in the tables that follow.

For the subsets of respondents involved in trial design or analysis, we compared their responses for desirability versus actual use of evidence synthesis. For each of the five aspects of trial design, we categorised each respondent who indicated they had been involved in trial design into one of the following: ‘used and think desirable’, ‘used but don’t think desirable’, ‘not used and don’t think desirable’ and ‘not used but think desirable’. For each of the three aspects of trial analysis, we added three categories to these options: ‘used and don’t know whether desirable’, ‘not used and don’t know whether desirable’ and ‘don’t understand’.

To summarise responses about the three greatest barriers to the use of evidence synthesis, we assigned three points to the first (greatest perceived) barrier, two to the second and one to the third for each respondent. If a respondent had ticked three barriers but not indicated a ranking, each was assigned two points. No points were allocated for respondents who did not answer the question. For each potential barrier, the scores were then summated across respondents, so that higher overall scores indicated greater perceived barriers.

Although highly exploratory in nature because of small numbers, we examined answers to specific questions for two subgroups: the perceived barriers to the use of evidence synthesis in practice by statisticians specifically, statisticians’ use versus perceived desirability of using evidence synthesis in trial analysis, and the views of health economists on VoI analyses.

## Results

There were 106 respondents, of whom 54 (51%) were statisticians, 8 (8%) were health economists and 18 (17%) worked in trial management. These are overlapping categories, i.e. respondents were asked to select all roles that applied to them. All respondents had spent some time working in the area of trials: 86 (81%) for at least 3 years and 32 (30%) for more than 10 years. Ninety-six (91%) respondents indicated that they had been involved in the design, setting up or running of trials (77 (80%) in a clinical trials unit and 9 (9%) in industry). Eighty-five (80%) indicated that they had been involved in trial design, 71 (67%) in trial conduct, 73 (69%) in statistical analysis and 52 (49%) had been involved in undertaking a systematic review of trials. Only three (3%) respondents indicated that they had not been involved in any of these. Full details are shown in Additional file 2: Table S1.

### The use of evidence synthesis to inform trial design

**Fig. 1:**
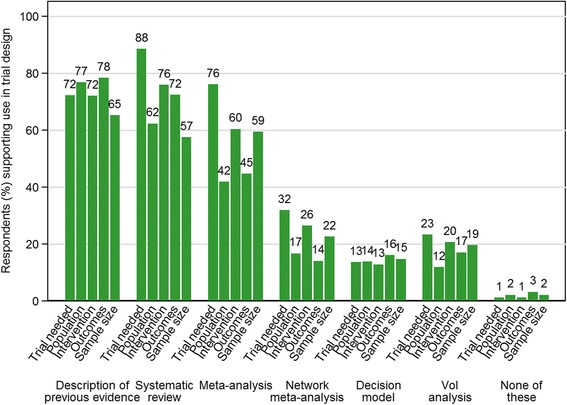
Views of respondents on whether evidence synthesis methods *should* be used to inform trial design. The type of evidence synthesis method is summarised across five aspects of trial design: whether a trial is needed (*n* = 104), choice of population (*n* = 103), choice of interventions (*n* = 103), choice of outcomes and follow-up time (*n* = 101), sample size (*n* = 103)

Figure 1 summarises the views of respondents on the desirability of using evidence synthesis in trial design. Support for using a description of previous evidence or a systematic review to inform each aspect listed was high. For most aspects of design, support was slightly higher for a simple description of previous evidence than a systematic review. In contrast, there was slightly more support for a systematic review to inform whether a trial is needed (92/104 or 89% systematic review versus 75/104 or 72% description of previous evidence) and the choice of interventions (78/103, 76% versus 74/103, 72%, respectively). Over 50% of respondents also felt that a meta-analysis should be used to inform whether a trial is needed, the choice of interventions and the sample size. Fewer respondents indicated support for the use of more complex analyses (NMA, decision models and VoI analyses). For example, only 19% (20/101 respondents) indicated that VoI analyses should be used to inform sample size calculations. Of these respondents, 55% (11/20 respondents) were statisticians and 20% (4/20 respondents) were health economists including one person who identified themselves in both roles. However, six of the eight health economists (75%) supported such use of VoI calculations across at least one aspect of design. All respondents indicated support for using some form of evidence synthesis in at least three of the five aspects of trial design that were listed. Seven respondents, all of whom had experience in trial design, suggested that no form of evidence synthesis was required for one or two specific aspects, most commonly ‘choice of outcomes and follow-up time’ (3/101 or 3% of respondents). Full results are shown in Additional file 3: Table S2.

**Fig. 2:**
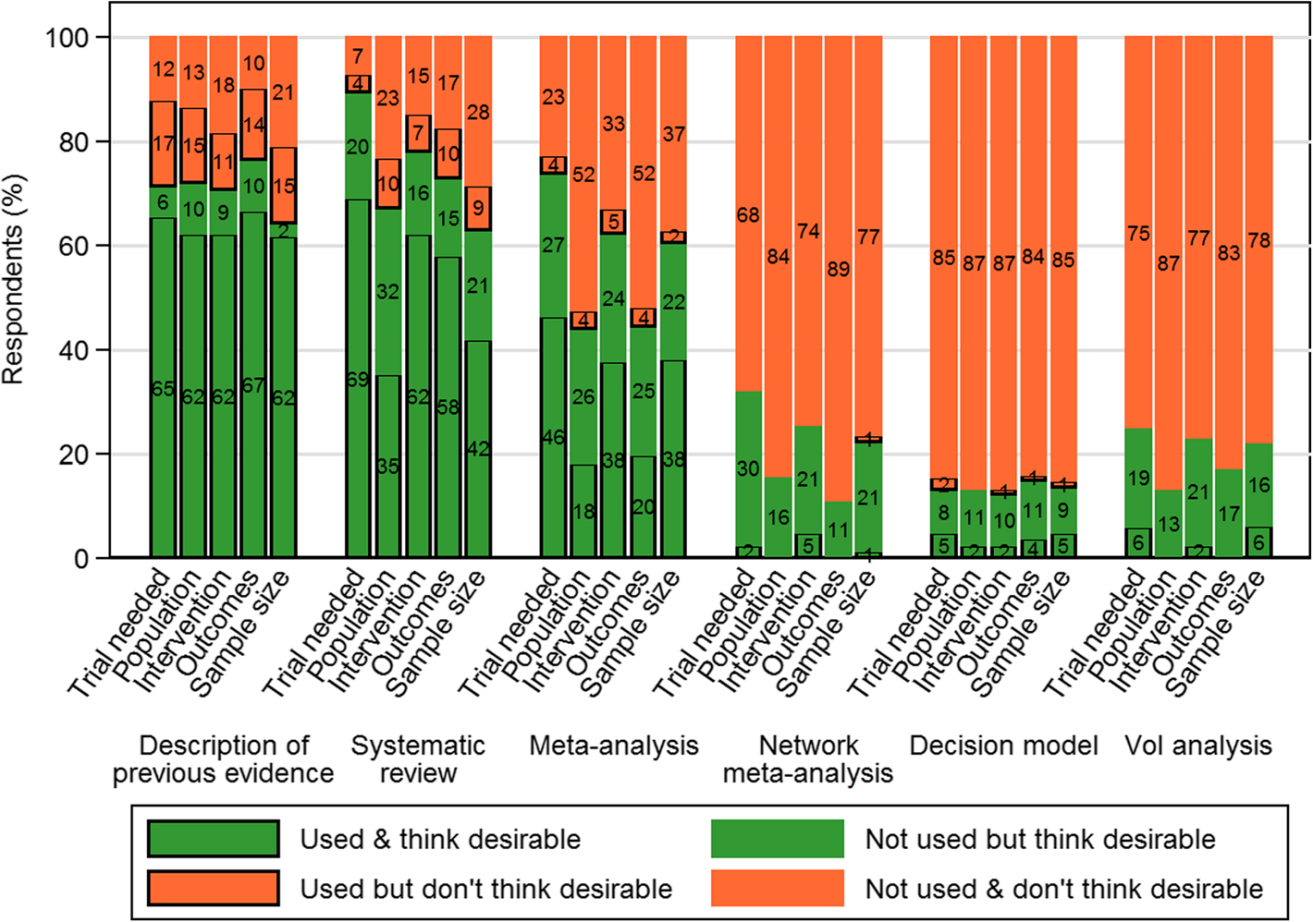
Comparisons between desirable and current practice in the use of evidence synthesis methods in trial design. This is summarised by type of evidence synthesis method, among survey respondents involved in trial design to inform five aspects of trial design: whether a trial is needed (*n* = 84), choice of population (*n* = 82), choice of interventions (*n* = 82), choice of outcomes and follow-up time (*n* = 81), sample size (*n* = 81). Numbers displayed are percentages

Of the 85 respondents who indicated involvement in trial design, Fig. 2 contrasts their views on how evidence synthesis methods should be used versus their own use during the last 10 years. Full results are shown in Additional file 3: Table S3. Slightly more respondents indicated that they had used a description of previous evidence to inform aspects of trial design than had indicated that such use was desirable. For example, 82% (69/84) had used a description of previous evidence to decide whether a trial is needed, compared with 71% (60/84) indicating support for such use. Of the 69 respondents who had used a description of previous evidence in this way, 14 (20%) did not indicate that such use was desirable. In contrast, our results suggested that trial design practitioners would like to be using each of the other five types of evidence synthesis more than they currently do in practice. This pattern was consistent across all aspects of trial design. For example, only 50% (42/84) of respondents had used a meta-analysis to inform whether a trial is needed, whereas 74% (62/84) thought that it was desirable. Ninety-three percent of those who had used a meta-analysis to inform whether a trial is needed (39/42) felt that such use was desirable. Some 96% (78/81) of respondents claimed to have used some form of evidence synthesis to inform sample size calculations in the last 10 years, close to the 99% (80/81) who indicated support for such use (data not shown). Making the same comparison but excluding the less formal ‘description of previous evidence’, we found a larger discrepancy: 62% (50/81) had used evidence synthesis methods to inform sample size calculations, compared with 84% (68/81) indicating that this is desirable (data not shown). Only 6% (5/81) of respondents had used a VoI analysis to inform sample size calculations, compared with 22% (18/81) indicating that VoI analysis should be used for this. All five respondents who had used VoI in this way were in support of its use. For all types of evidence synthesis methods except VoI analyses, which was mostly conducted by the clinical trials team, the use of previously published evidence syntheses was most common (see Additional file 3: Table S4).

### The use of evidence synthesis to inform trial analysis

**Fig. 3:**
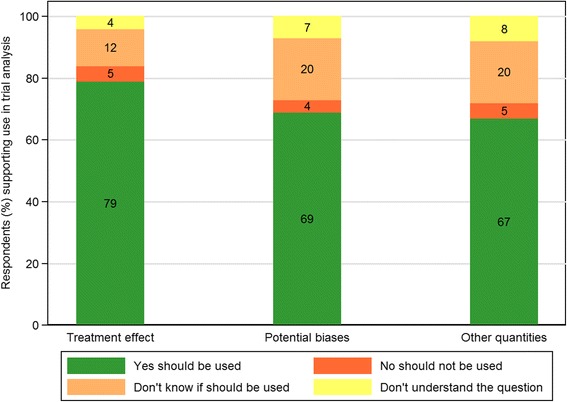
Views of respondents on whether evidence synthesis *should* be used to inform trial analysis. This is summarised across three aspects of trial analysis: the treatment effect, potential biases arising from trial conduct and other quantities (of *n* = 100 people who answered this question)

Seventy-nine percent (79/100) of respondents indicated that external information about the treatment effect should be used to inform aspects of the analysis (see Fig. 3; Additional file 4: Table S5). Similarly, 69% (69/100) expressed support for using external information related to potential biases in trial analysis and 67% (67/100) for the use of external evidence on other quantities which are usually poorly estimated. While only a few respondents (5% or less) indicated that external evidence should not be used in these ways, between 15 and 30% selected the ‘don’t know’ or ‘don’t understand’ options.

**Fig. 4:**
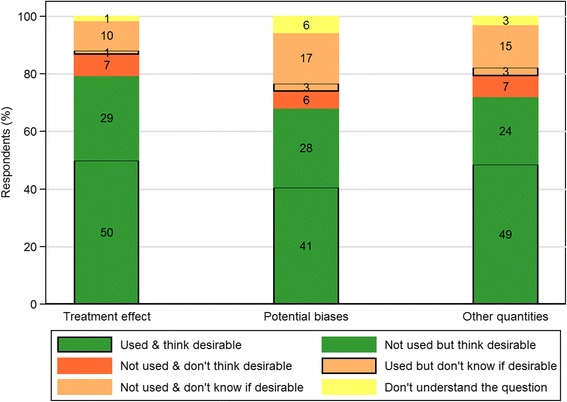
Comparisons between desirable and current practice in the use of evidence synthesis methods in trial analysis. This is summarised among survey respondents involved in trial analysis to inform three aspects of trial analysis: the treatment effect (*n* = 68), potential biases arising from trial conduct (*n* = 69) and other quantities (*n* = 68). Numbers displayed as percentages

Seventy-three out of one hundred and six (69%) respondents were involved in trial analysis. Figure 4 contrasts the views of this subsample on how evidence synthesis methods should be used to inform aspects of analysis versus their own use in practice. 52% (35/68) indicated that, during the past 10 years, they had used external information about the treatment effect to inform trial analysis, compared with 79% (54/68) indicating support for such use. 97% of those who had used external information in this way (34/35) felt that such use was desirable. While 63% (20/32) of respondents who had *not* used external information about the treatment effect in trial analysis also felt such use was desirable, 22% (7/32) were not sure. Similar patterns were seen for using external evidence on potential biases and other quantities. Full results are shown in Additional file 4: Table S6. A sensitivity analysis including only statisticians suggested slightly *less* use of external evidence in each of the three areas (see Additional file 4: Figure S1).

### Barriers to the use of evidence synthesis methods

**Fig. 5:**
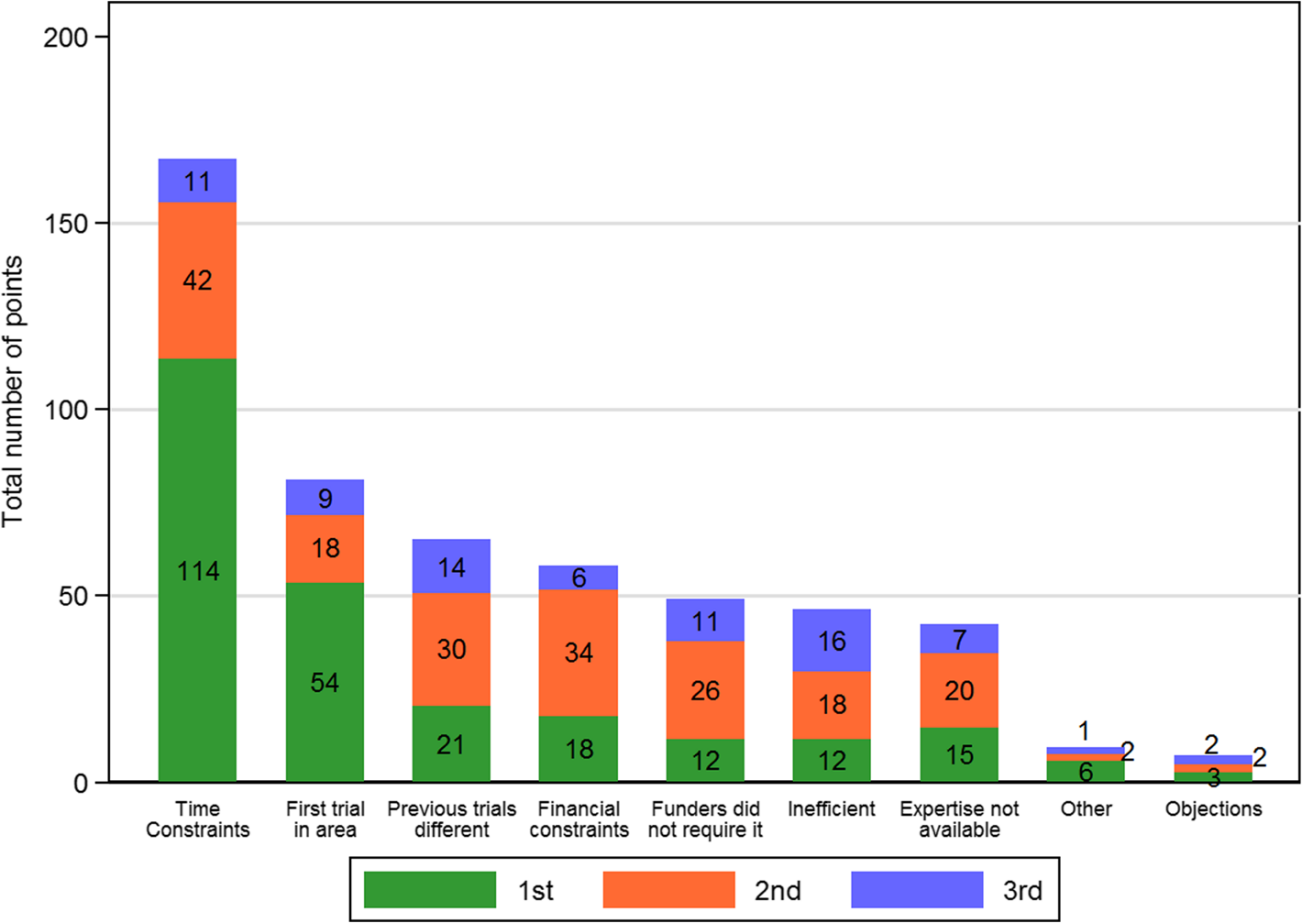
Barriers to the use of evidence synthesis (higher scores indicate greatest perceived barriers). Three points were assigned to the greatest barrier, 2 points to the second and 1 to the third. For example, 38 respondents ranked time constraints as the greatest barrier (3 × 38 = 114 points), 21 ranked it second (2 × 21 = 42) and 11 ranked it third (1 × 11 = 11)

Figure 5 shows the barriers to using evidence synthesis, ordered by their perceived importance. The bars show the total number of points awarded to each barrier, split by the number of points it acquired by being ranked the first, second and third greatest barrier. 87% (90/103) of respondents answered this question. By far the greatest perceived barrier was time constraints. This was followed by a belief that the trial was the first in the area and a belief that previous trials were different from the current trial. Of those selecting ‘other’, reasons included complexity of the trials and the ‘chief investigator had more evidence than previously published information.’ ‘Objections to using evidence syntheses (from you or colleagues)’ was the lowest scoring barrier of those listed. The conclusions remained unchanged when the analysis was restricted to statisticians only (data not shown).

## Discussion

Our INVEST survey indicates a high level of support for the use of evidence synthesis to inform aspects of trial design and analysis. Support was generally high for using a description of previous evidence, a systematic review or a meta-analysis when designing a trial. Fewer respondents indicated support for the use of NMA, decision models and VoI analyses. Only a few respondents (approximately 5%) felt that external evidence about particular parameters should not be used in the analysis of a trial; however, many (up to 20%) did not know if such evidence should be used in practice. Our results indicate some discrepancies between the evidence synthesis methods that people think should be used and what they are using in current practice. In particular, respondents did not appear to be using systematic reviews, meta-analyses, NMAs, decision models and VoI analyses as much as they wanted across all aspects of trial design. The greatest perceived barrier to using evidence synthesis methods in trial design or analysis was time constraints, followed by a belief that the new trial was the first in the area.

The sampling frame was approximately 638 people, but only 106 completed the survey, providing a response rate of approximately 17% and a potential for selection bias. We were unable to obtain information on the characteristics of the nonrespondents which would have enabled us to explore the representativeness of our sample, but it is possible that respondents were more enthusiastic about evidence synthesis methods than nonrespondents. Some 95% of our sampling frame were from the UK and the Republic of Ireland, so the results may not be generalisable to the international clinical trials community. Further, our sampling frame consisted of conference delegates closely involved in trial design and analysis, who are likely to have a strong interest in promoting good practice. As such, we might expect our sample to answer some of the questions more favourably than the wider population of people involved in clinical trials. In particular, half of respondents were statisticians (51%), who may be expected to be more open to advanced statistical methods (such as using evidence syntheses to improve precision in estimates of some parameters) compared with other contributors to the design, conduct or delivery of trials. Statisticians are also influential members of the multidisciplinary teams that are involved in trial design and may be useful advocates for the increased use of available evidence in trial design. Although it would have been interesting to explore differences across research centres and countries, we chose not to collect such geographical data to protect anonymity and minimise the burden of survey completion. To summarise the barriers to the use of evidence synthesis, we assigned scores based on an arbitrary assumption of linearity, i.e. such that an individual’s highest ranked barrier is three times as important as their third barrier. These scores, although helpful for summarising data, might not reflect respondents’ true views. We intended all listed barriers to be interpreted as reasons why a trial team might not seek or carry out evidence synthesis. However, it is possible that some respondents who chose ‘Believed to be the first trial in the area’ could have been thinking of the situation where a literature search or systematic review reveals no previous trials. The extent of this barrier would then be overestimated.

In trial design, for both whether a trial was needed and for choosing an intervention, more respondents said that a systematic review, rather than a less formal description of previous evidence, should be used. It therefore seems that respondents felt the need for a thorough, systematic approach in order to show convincingly whether there is a gap in the evidence base that merits a new trial. For the other aspects of trial design, there may not be sufficient available evidence to warrant a systematic review, so that a less formal description of previous evidence might be felt to be adequate.

The large proportions of respondents who indicated that they had either used evidence synthesis to inform trial analysis or that they believed evidence synthesis should be used in this way were surprising. Even more surprisingly, a sensitivity analysis including only statisticians provided slightly *lower* estimates of these proportions, although the small sample size precludes strong assertions. We feel that it is unlikely that these relatively advanced methods are being used so frequently in practice. As such, we suggest that many respondents may have interpreted these questions in ways other than intended. This explanation appears to be supported by the result that fewer statisticians than nonstatisticians claim to be using external evidence in this way: it is likely that confusion about these questions was higher among nonstatisticians although we have no direct evidence of this. In particular, respondents might have interpreted the incorporation of ‘external information about the treatment effect (including a meta-analysis)’ in trial analysis as meaning including the new trial results in an updated meta-analysis. Our intention had instead been to elicit views on the use of informative prior distributions in a Bayesian statistical framework. In retrospect, we should have clarified these questions about relatively complex issues using examples, although we were keen to be as concise as possible. We propose that future qualitative research should be conducted to explore the use of informative priors with particular focus on evidence about the treatment effect, potential biases and other quantities in trial analysis [19]. This work should investigate more thoroughly how trialists are currently using evidence synthesis to inform analysis, and the potential barriers to an increased amount of such use. We would anticipate more objections in principle to the use of informative prior distributions compared with less formal uses of evidence synthesis. The qualitative work should explore which types of external evidence might be considered most relevant and useful to trial analysis, and what level of such use might be acceptable in practice.

Funders of clinical trials often highlight the importance of taking into account existing evidence in grant applications [20]. However, it is still unclear how, and to what extent, funders or reviewers expect evidence synthesis to be used. We did not explore the views of funders or reviewers specifically but this could be another valuable avenue for future research, given the critical role that they could play in minimising research wastage.

The INVEST survey provides generally higher estimates of the use of systematic reviews in trial design than the recent review of Bhurke et al. [7], with the exception of ‘justification of the trial’ (Bhurke et al. 94% versus INVEST 73%). For example, 68% of our respondents indicated that they had used a systematic review to inform choice of outcomes and follow-up time, whereas only 16% and 6% of trials reviewed by Bhurke et al. had used a review to inform these two aspects, respectively. Similarly, 51% of our respondents said that they had used a systematic review to inform sample size calculations, seemingly in contrast to the finding of Bhurke et al. that only 9% of trials had used a review to inform the standard deviation and 3% to ‘estimate the difference to detect or margin of equivalence’. It is possible that other trials in the Bhurke et al. review relied on pilot trials to inform these parameters [21, 22], while the INVEST results seem to suggest that relevant information will often be available from evidence syntheses. However, the results are not directly comparable since we asked respondents to consider all trials that they had been involved in during the last 10 years, whereas Bhurke et al. investigated whether evidence synthesis had been used in specific individual trials. On the other hand, Bhurke et al. reviewed only publicly funded (NIHR HTA) trials, while trialists attending ICTMC are likely to also participate in company-funded trials, for which less justification is required and there is possibly a stronger expectation for independently clear results. In agreement with Bhurke et al. we found that important barriers to the use of evidence synthesis in practice include a new trial being the first in its area or being different from trials included in a previous review. However, by directly asking trialists instead of relying on documentation, we were able to see that the greatest barrier is time constraints. In attempt to overcome the issue of time constraints when synthesising evidence, many methods for rapid reviews have been proposed over recent years [23, 24]. Khangura et al. [23] developed their own eight-step approach of conducting a rapid review having reviewed the current literature. Implementation of their approach in HTA trials has been successful and can be applied to other types of trials [25]. However, more training on approximate methods and rapid reviews is needed to support their wider use in practice. Investment in adequate resources and training at this stage could lead to cost savings in the longer term, by reducing waste in research.

We found less support for the use of NMAs, decision models and VoI analyses in trial design which may be because they are more complex to conduct and require a greater investment of time and expertise. These methods could further help inform decisions but also require additional assumptions and ‘a priori’ parameter estimates, such as the cost-effectiveness threshold and parameters related to structural uncertainties in the case of VoI, which may not be available. A policy framework on when, and how, to perform such analyses and how they are used could be a useful next step [26]. We also note that most individual trials investigate a specific research question for one particular treatment: for example, in 2014, 80% of trials were still two-armed trials [27]. In contrast, NMAs, decision models and VoI analyses are commonly used to make decisions and inform policy when there is a choice between a number of concurrent treatment options. These methods could be considered less relevant in the design and analysis of an individual two-armed trial. VoI analyses, in particular, are usually commissioned in high-value trials, often in situations with many treatments and uncertainty as to which is best. However, a NMA could be more relevant to inform the interventions of a two-armed trial if used at the earlier part of the design process [28]. Trial-based economic analysis are sometimes secondary to the clinical aspect rather than being fully integrated within a trial design [29] meaning that the use of decision models and VoI analyses to inform trial design is limited. Only 6% (5/84) of our respondents had used a VoI analysis to inform whether a trial is needed, although all of those who had used a VoI analysis were in favour of its use more generally. Models in health economic analyses are a strongly simplified representation of disease history and treatment effects and are framed around a particular decision setting (e.g. UK) using setting-specific values for health care use, costs and health benefits. These values may change over time and are likely to be different in other settings. Streamlining of decision modelling and VoI analyses would, therefore, be particularly challenging. Despite the recognition that the VoI method does come with its assumptions and limitations, its potential to guide the need for and the design of new studies [30] warrant its wider consideration and further development.

## Conclusions

Trial teams responding to the INVEST survey generally reported that they are using evidence synthesis in trial design and analysis more than we might have expected, but less than they might like to. Time constraints was identified as the greatest barrier to more widespread use. Further research on ways to undertake evidence synthesis more efficiently, and training on how to incorporate results from these into existing procedures will help to ensure the best use of relevant external evidence in the design, conduct and analysis of clinical trials.

## Additional files

Additional file 1: Shows the INVEST survey. (DOCX 130 kb)

Additional file 2: Shows the characteristics of respondents. (DOCX 14 kb)

Additional file 3: Shows tables summarising desirable and current use of evidence synthesis to inform trial design. (DOCX 20 kb)

Additional file 4: Shows tables summarising desirable and current the use of evidence synthesis to inform trial analysis. (DOCX 3086 kb)

## Abbreviations

HTA: Health Technology Assessment
ICTMC: International Clinical Trials Methodology Conference
INVEST: INVestigating the use of Evidence Synthesis in the design and analysis of clinical Trials
NIHR: National Institute for Health Research
NMA: Network meta-analysis
VoI: Value of information
