# Supplementary material for: The INVEST project: investigating the use of evidence synthesis in the design and analysis of clinical trials
Source: Trials. 2017 May 15;18:219. doi: 10.1186/s13063-017-1955-y (PMC5433067; doi:10.1186/s13063-017-1955-y)
Supplement: Supplementary file 2 — Shows the characteristics of respondents. (DOCX 14 kb) [file 13063_2017_1955_MOESM2_ESM.docx]

**Table 1: Respondent characteristics**

| N=106 | | | n | % |
| --- | --- | --- | --- | --- |
| ***Job/role**** | | | | |
| Clinician | | | 12 | 11.3 |
| Clinical co-ordinator | | | 1 | 0.9 |
| Data management | | | 5 | 4.7 |
| Epidemiologist | | | 5 | 4.7 |
| Health economist | | | 8 | 7.5 |
| Information specialist | | | 2 | 1.9 |
| Programmer | | | 1 | 0.9 |
| Qualitative researcher | | | 10 | 9.4 |
| Statistician | | | 54 | 50.9 |
| Student▪: | | | 10 | 9.4 |
|  | | Clinician | 1/10 |  |
|  | | Epidemiologist | 1/10 |  |
|  | | Health services | 1/10 |  |
|  | | Statistician | 4/10 |  |
|  | | Statistician and data manager | 1/10 |  |
|  | | Unspecified | 2/10 |  |
| Trial management | | | 18 | 17.0 |
| Other^: | | | 15 | 14.2 |
|  | | Academic researcher | 4/15 |  |
|  | | Chief/principal investigators | 4/15 |  |
|  | | Director of trials unit/CEO | 2/15 |  |
|  | | Network coordinator | 1/15 |  |
|  | | Research Funder | 1/15 |  |
|  | | Systematic reviewer | 1/15 |  |
|  | | Trial methodologist | 3/15 |  |
| ***Involved in design, setting up or running trials in your job/role*** | | | | |
| None at all | | | 10 | 9.4 |
| Clinical trials unit only | | | 59 | 55.7 |
| Industry only | | | 1 | 0.9 |
| Clinical trials unit and Industry | | | 6 | 5.7 |
| Clinical trials unit and other setting: | | | 12 | 11.3 |
|  | CTU + Academia | | 10/12 |  |
|  | CTU + NHS/Hospital | | 2/12 |  |
| Industry and other setting: | | | 2 | 1.9 |
|  | MRC unit | | 2/2 |  |
| Other: | | | 16 | 15.1 |
|  | Academia | | 9/16 |  |
|  | NHS/Hospital | | 4/16 |  |
|  | Academia/Hospital | | 3/16 |  |
| Involved in a trials unit at some point | | | 77 | 72.6 |
| ***Time spent working in the area of clinical trials*** | | | | |
| Not at all | | | 0 | 0 |
| 0-2 years | | | 20 | 18.9 |
| 3-5 years | | | 30 | 28.3 |
| 6-10 years | | | 14 | 13.2 |
| 11-20 years | | | 26 | 24.5 |
| Over 20 years | | | 16 | 15.1 |
| ***Aspects of clinical trials have you been involved in*** | | | | |
| Trial design | | | 85 | 80.2 |
| Trial conduct | | | 71 | 67.0 |
| Statistical analysis | | | 73 | 68.9 |
| Undertaking a systematic review of trials | | | 52 | 49.1 |
| None of these | | | 3 | 2.8 |

*This question was ‘tick all that apply’ so respondents could have selected more than 1 and therefore the percentages do not add up to 100%

▪Student disciplines will have already been counted if they ticked one of the available options

^Could also have ticked any of the available options
