# Supplementary material for: The INVEST project: investigating the use of evidence synthesis in the design and analysis of clinical trials
Source: Trials. 2017 May 15;18:219. doi: 10.1186/s13063-017-1955-y (PMC5433067; doi:10.1186/s13063-017-1955-y)
Supplement: Supplementary file 3 — Shows tables summarising desirable and current use of evidence synthesis to inform trial design. (DOCX 20 kb) [file 13063_2017_1955_MOESM3_ESM.docx]

**Table 1: How evidence synthesis ‘should’ be used in trial design**

| N=106 | Description of previous evidence | | Systematic review | | Meta-analysis | | Network meta-analysis | | Decision model | | VoI analysis | | None of  these | |
| --- | --- | --- | --- | --- | --- | --- | --- | --- | --- | --- | --- | --- | --- | --- |
|  | n | % | n | % | n | % | n | % | n | % | n | % | n | % |
| Whether a trial is needed* | 75 | 72.1 | 92 | 88.5 | 79 | 76.0 | 33 | 31.7 | 14 | 13.5 | 24 | 23.1 | 1 | 1.0 |
| Choice of population** | 79 | 76.7 | 64 | 62.1 | 43 | 41.7 | 17 | 16.5 | 14 | 13.6 | 12 | 11.7 | 2 | 1.9 |
| Choice of interventions× | 74 | 71.8 | 78 | 75.7 | 62 | 60.2 | 27 | 26.2 | 13 | 12.6 | 21 | 20.4 | 1 | 1.0 |
| Choice of outcomes and follow-up time×× | 79 | 78.2 | 73 | 72.3 | 45 | 44.6 | 14 | 13.9 | 16 | 15.8 | 17 | 16.8 | 3 | 3.0 |
| Sample size▪ | 67 | 65.0 | 59 | 57.3 | 61 | 59.2 | 23 | 22.3 | 15 | 14.6 | 20 | 19.4 | 2 | 1.9 |
| Any design aspect▪▪ | 88 | 87.1 | 93 | 92.1 | 87 | 86.1 | 38 | 37.6 | 28 | 27.7 | 35 | 34.7 | 7 | 6.9 |

*2 respondents with missing data, **3 respondents with missing data, ×3 respondents with missing data, ××5 respondents with missing data, ▪3 respondents with missing data, ▪▪5 respondents with missing data.

**Table 2: Actual uses of evidence synthesis in trial design during the last 10 years, compared with uses considered desirable**

| **N=85** | **Yes used, yes desirable** | | **Not used, yes desirable** | | **Not used, not desirable** | | **Yes used, not desirable** | | **Total used** | | **Total *should* be used** | |
| --- | --- | --- | --- | --- | --- | --- | --- | --- | --- | --- | --- | --- |
|  | **n** | **%** | **n** | **%** | **n** | **%** | **n** | **%** | **n** | **%** | **n** | **%** |
| **Whether a trial is needed*** |  |  |  |  |  |  |  |  |  |  |  |  |
| Description of previous evidence | 55 | 65.5 | 5 | 6.0 | 10 | 11.9 | 14 | 16.7 | 69 | 82.2 | 60 | 71.5 |
| Systematic review | 58 | 69.0 | 17 | 20.2 | 6 | 7.1 | 3 | 3.6 | 61 | 72.6 | 75 | 89.2 |
| Meta-analysis | 39 | 46.4 | 23 | 27.4 | 19 | 22.6 | 3 | 3.6 | 42 | 50 | 62 | 73.8 |
| Network meta-analysis | 2 | 2.4 | 25 | 29.8 | 57 | 67.9 | 0 | 0.0 | 2 | 2.4 | 27 | 32.2 |
| Decision model | 4 | 4.8 | 7 | 8.3 | 71 | 84.5 | 2 | 2.4 | 6 | 7.2 | 11 | 13.1 |
| VoI analysis | 5 | 6.0 | 16 | 19.0 | 63 | 75.0 | 0 | 0.0 | 5 | 6 | 21 | 25 |
| **Choice of population**× |  |  |  |  |  |  |  |  |  |  |  |  |
| Description of previous evidence | 51 | 62.2 | 8 | 9.8 | 11 | 13.4 | 12 | 14.6 | 63 | 76.8 | 59 | 72 |
| Systematic review | 29 | 35.4 | 26 | 31.7 | 19 | 23.2 | 8 | 9.8 | 37 | 45.2 | 55 | 67.1 |
| Meta-analysis | 15 | 18.3 | 21 | 25.6 | 43 | 52.4 | 3 | 3.7 | 18 | 22 | 36 | 43.9 |
| Network meta-analysis | 0 | 0.0 | 13 | 15.9 | 69 | 84.1 | 0 | 0.0 | 0 | 0 | 13 | 15.9 |
| Decision model | 2 | 2.4 | 9 | 11.0 | 71 | 86.6 | 0 | 0.0 | 2 | 2.4 | 11 | 13.4 |
| VoI analysis | 0 | 0.0 | 11 | 13.4 | 71 | 86.6 | 0 | 0.0 | 0 | 0 | 11 | 13.4 |
| **Choice of interventions**▪ |  |  |  |  |  |  |  |  |  |  |  |  |
| Description of previous evidence | 51 | 62.2 | 7 | 8.5 | 15 | 18.3 | 9 | 11.0 | 60 | 73.2 | 58 | 70.7 |
| Systematic review | 51 | 62.2 | 13 | 15.9 | 12 | 14.6 | 6 | 7.3 | 57 | 69.5 | 64 | 78.1 |
| Meta-analysis | 31 | 37.8 | 20 | 24.4 | 27 | 32.9 | 4 | 4.9 | 35 | 42.7 | 51 | 62.2 |
| Network meta-analysis | 4 | 4.9 | 17 | 20.7 | 61 | 74.4 | 0 | 0.0 | 4 | 4.9 | 21 | 25.6 |
| Decision model | 2 | 2.4 | 8 | 9.8 | 71 | 86.6 | 1 | 1.2 | 3 | 3.6 | 10 | 12.2 |
| VoI analysis | 2 | 2.4 | 17 | 20.7 | 63 | 76.8 | 0 | 0.0 | 2 | 2.4 | 19 | 23.1 |
| **Choice of outcomes and follow-up time**◦ |  |  |  |  |  |  |  |  |  |  |  |  |
| Description of previous evidence | 54 | 66.7 | 8 | 9.9 | 8 | 9.9 | 11 | 13.6 | 65 | 80.3 | 62 | 76.6 |
| Systematic review | 47 | 58.0 | 12 | 14.8 | 14 | 17.3 | 8 | 9.9 | 55 | 67.9 | 59 | 72.8 |
| Meta-analysis | 16 | 19.8 | 20 | 24.7 | 42 | 51.9 | 3 | 3.7 | 19 | 23.5 | 36 | 44.5 |
| Network meta-analysis | 0 | 0.0 | 9 | 11.1 | 72 | 88.9 | 0 | 0.0 | 0 | 0 | 9 | 11.1 |
| Decision model | 3 | 3.7 | 9 | 11.1 | 68 | 84.0 | 1 | 1.2 | 4 | 4.9 | 12 | 14.8 |
| VoI analysis | 0 | 0.0 | 14 | 17.3 | 67 | 82.7 | 0 | 0.0 | 0 | 0 | 14 | 17.3 |
| **Sample size**¥ |  |  |  |  |  |  |  |  |  |  |  |  |
| Description of previous evidence | 50 | 61.7 | 2 | 2.5 | 17 | 21.0 | 12 | 14.8 | 62 | 76.5 | 52 | 64.2 |
| Systematic review | 34 | 42.0 | 17 | 21.0 | 23 | 28.4 | 7 | 8.6 | 41 | 50.6 | 51 | 63 |
| Meta-analysis | 31 | 38.3 | 18 | 22.2 | 30 | 37.0 | 2 | 2.5 | 33 | 40.8 | 49 | 60.5 |
| Network meta-analysis | 1 | 1.2 | 17 | 21.0 | 62 | 76.5 | 1 | 1.2 | 2 | 2.4 | 18 | 22.2 |
| Decision model | 4 | 4.9 | 7 | 8.6 | 69 | 85.2 | 1 | 1.2 | 5 | 6.1 | 11 | 13.5 |
| VoI analysis | 5 | 6.2 | 13 | 16.0 | 63 | 77.8 | 0 | 0.0 | 5 | 6.2 | 18 | 22.2 |

*1 respondent with missing data, ×3 respondents with missing data , ▪3 respondents with missing data, ◦4 respondents with missing data, ¥4 respondents with missing data.

**Table 3: Types of evidence synthesis used in trial design**

| N=85 | Description of previous evidence | | Systematic review | | Meta-analysis | | Network meta-analysis | | Decision model | | VoI analysis | |
| --- | --- | --- | --- | --- | --- | --- | --- | --- | --- | --- | --- | --- |
|  | n | % | n | % | n | % | n | % | n | % | n | % |
| Previously published evidence syntheses | 62 | 72.9 | 59 | 69.4 | 46 | 54.1 | 5 | 5.9 | 7 | 8.2 | 1 | 1.2 |
| Conducted by the clinical trial team | 59 | 69.4 | 55 | 64.7 | 31 | 36.5 | 2 | 2.4 | 7 | 8.2 | 6 | 7.1 |
