# Supplementary material for: The INVEST project: investigating the use of evidence synthesis in the design and analysis of clinical trials
Source: Trials. 2017 May 15;18:219. doi: 10.1186/s13063-017-1955-y (PMC5433067; doi:10.1186/s13063-017-1955-y)

**Table 1: How evidence synthesis methods ‘should’ be used to inform aspects of trial analysis**

| N=100 | Yes | | No | | Don’t know | | I don’t understand | |
| --- | --- | --- | --- | --- | --- | --- | --- | --- |
|  | n | % | n | % | n | % | n | % |
| External information about the treatment effect (including a meta-analysis) | 79 | 79.0 | 5 | 5.0 | 12 | 12.0 | 4 | 4.0 |
| External information related to potential biases arising from trial conduct (e.g. blinding infeasible) | 69 | 69.0 | 4 | 4.0 | 20 | 20.0 | 7 | 7.0 |
| External information about other quantities involved in the analysis (e.g. correlations or baseline event rates) | 67 | 67.0 | 5 | 5.0 | 20 | 20.0 | 8 | 8.0 |

**Table 2: Actual uses of evidence synthesis in trial analysis during the last 10 years, compared with uses considered desirable**

| N=73 | Yes used, yes desirable | | Not used, yes desirable | | Not used, not desirable | | Yes used, not sure | | Yes used, not desirable | | Not used, not sure | | Don't understand | |
| --- | --- | --- | --- | --- | --- | --- | --- | --- | --- | --- | --- | --- | --- | --- |
|  | n | % | n | % | n | % | n | % | n | % | n | % | n | % |
| External information about the treatment effect(including a meta-analysis) * | 34 | 50.0 | 20 | 29.4 | 5 | 7.4 | 1 | 1.5 | 0 | 0 | 7 | 10.3 | 1 | 1.5 |
| External information related to potential biases arising from trial conduct (e.g. blinding infeasible) × | 28 | 40.6 | 19 | 27.5 | 4 | 5.8 | 2 | 2.9 | 0 | 0 | 12 | 17.4 | 4 | 5.8 |
| External information about other quantities involved in the analysis (e.g. correlations or baseline event rates) ▪ | 33 | 48.5 | 16 | 23.5 | 5 | 7.4 | 2 | 2.9 | 0 | 0 | 10 | 14.7 | 2 | 2.9 |

*5 respondents with missing data, ×4 respondents with missing data, ▪5 respondents with missing data.

**Figure 1: Comparisons between ideals and current practice of evidence synthesis methods among statisticians only in use of evidence synthesis methods to inform three different aspects of trial analysis: the treatment effect (n=54), potential biases arising from trial conduct (n=54) and other quantities (n=54)**


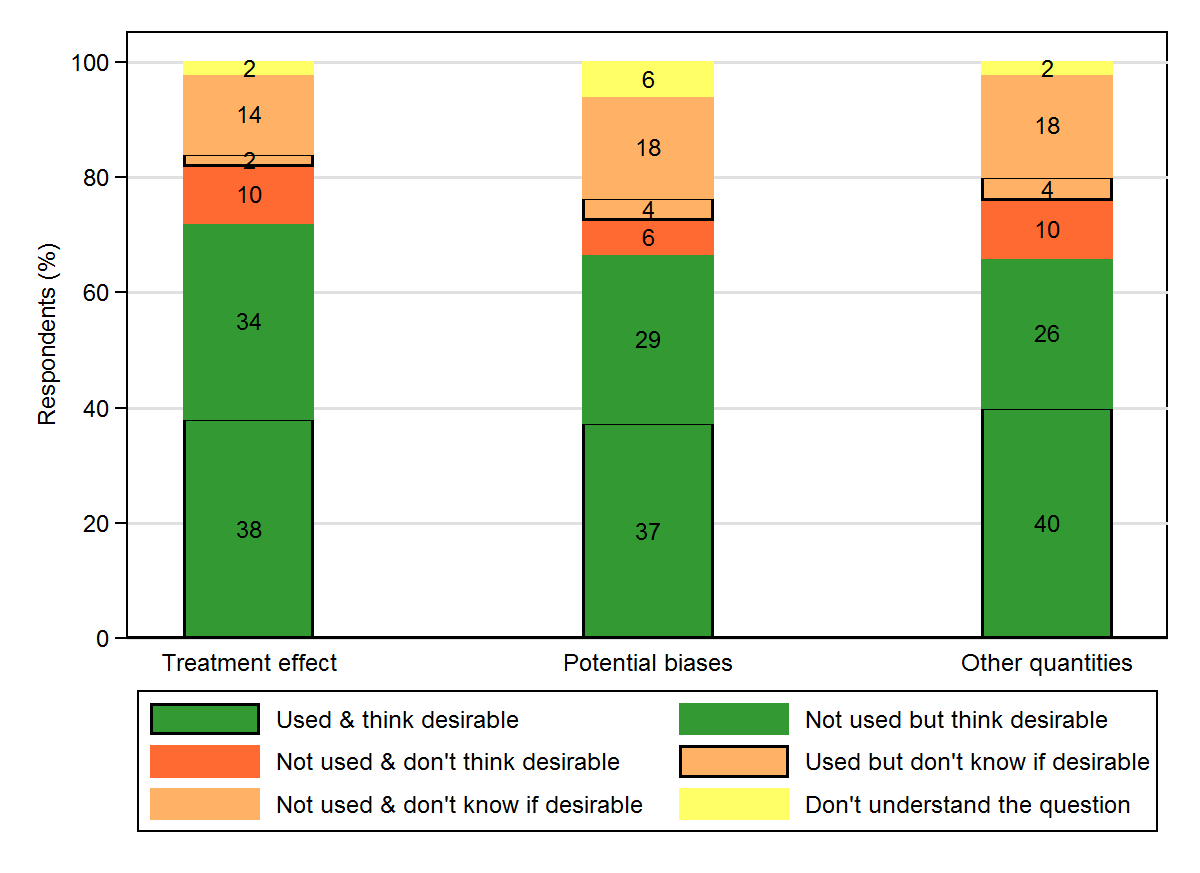

Supplement: Supplementary file 4 — Shows tables summarising desirable and current the use of evidence synthesis to inform trial analysis. (DOCX 3086 kb) [file 13063_2017_1955_MOESM4_ESM.docx]
