# Supplementary material for: The INVEST project: investigating the use of evidence synthesis in the design and analysis of clinical trials
Source: Trials. 2017 May 15;18:219. doi: 10.1186/s13063-017-1955-y (PMC5433067; doi:10.1186/s13063-017-1955-y)
Supplement: Supplementary file 4 [file 13063_2017_1955_MOESM1_ESM.docx]

The INVEST project

INVESTIGATING THE USE OF EVIDENCE SYNTHESIS IN THE DESIGN OF CLINICAL TRIALS

MRC Hubs for Trials Methodology Research

Please complete this survey at
bit.ly/investsurvey

This survey aims to investigate the current use of evidence synthesis by clinical trialists, and reasons why it might n[illegible] in practice.

**Definitions**	*Evidence synthesis involves the combination of multiple sources of evidence. This includes:*
Description of previous evidence	Conducting a literature review (systematic or otherwise) and summarising the findings.
Systematic review	A review to collate all evidence that fits pre-specified eligibility criteria in order to address a s[illegible] research question.
Meta-analysis	Statistically combining results from two or more studies addressing the same research ques[illegible]
Network meta-analysis	An extension of meta-analysis to allow the simultaneous comparison of the effectiveness of [illegible] interventions through the use of direct and indirect evidence.
Decision model	A model to allow the synthesis of all available sources of evidence into a single coherent an[illegible] model that can then be used to evaluate alternative policies.
Value of information (VoI) analysis	An analytical modelling framework (usually based on a decision model) used to assess whe[illegible] is value in conducting a new trial, and to identify the optimal design for such a trial.

1. **What is your job/role?** Tick all that apply.

☐ Clinician ☐ Clinical co-ordinator ☐ Data management ☐ Information specia[illegible]
☐ Qualitative researcher ☐ Trial management ☐ Epidemiologist ☐ Research nurse
☐ Health economist ☐ Statistician ☐ Programmer ☐ Student*
☐ Other, please specify: ____________________

**If you are a student please tick your specialty as well.*

2. **Have you been involved in design, setting up or running trials in your job/role?** Tick all that apply.

☐ Not at all ☐ In a clinical trials unit ☐ In industry ☐ In a different setting, please specify: ____

3. **How long have you spent working in the area of clinical trials?**

☐ Not at all ☐ 0 - 2 years ☐ 3 – 5 years ☐ 6 - 10 years ☐ 11 – 20 years ☐ Over 2[illegible]

4. **Which of the following aspects of clinical trials have you been involved in?** Tick all that apply.

☐ Trial design ☐ Trial conduct ☐ Statistical analysis
☐ Undertaking a systematic review of trials ☐ None of these

Answer questions 5 and 6 only if you have been involved in clinical trial design, otherwise go to question 7.

5. **Thinking about clinical trials in which you have been involved over the last 10 years, which of the followi[illegible] evidence synthesis (along the top) <u>have informed</u> particular aspects of trial design (down the left)? This i[illegible] using previously published evidence syntheses.** Tick all that apply.

	Description of previous evidence	Systematic review	Meta-analysis	Network meta-analysis	Decision model	VoI analysis
Whether a trial is needed	☐	☐	☐	☐	☐	☐
Choice of population	☐	☐	☐	☐	☐	☐
Choice of interventions	☐	☐	☐	☐	☐	☐
Choice of outcomes						

6. **If you indicated any use of evidence synthesis in question 5, then how were these undertaken?** Tick all that apply.

	Description of previous evidence	Systematic review	Meta-analysis	Network meta-analysis	Decision model	VoI analysis
Previously published evidence syntheses	☐	☐	☐	☐	☐	☐
Conducted by the clinical trial team	☐	☐	☐	☐	☐	☐

7. **Which of the following types of evidence synthesis do you think <u>should be used</u> to inform particular aspects of trial design?** Tick all that apply.

	Description of previous evidence	Systematic review	Meta-analysis	Network meta-analysis	Decision model	VoI analysis	None of these
Whether a trial is needed	☐	☐	☐	☐	☐	☐	☐
Choice of population	☐	☐	☐	☐	☐	☐	☐
Choice of interventions	☐	☐	☐	☐	☐	☐	☐
Choice of outcomes and follow-up time	☐	☐	☐	☐	☐	☐	☐
Sample size	☐	☐	☐	☐	☐	☐	☐

8. **Answer only if you have been involved in clinical trials. Please <u>rank the TOP 3</u> of the following potential reasons why evidence syntheses have not been used to inform clinical trials in which you have been involved, 1 being the biggest reason, 3 being the third biggest reason.**

Time constraints	☐
Expertise not available	☐
Financial constraints	☐
Inefficient – a lot of time and resources for little return	☐
Believed to be the first trial in the area	☐
Previous trials were different from the new trial	☐
Funders did not require it	☐
Objections to using evidence syntheses (from you or colleagues)	☐
Other (please specify) ………………………………………………………..	☐

9. **Evidence synthesis might also be used to inform aspects of <u>trial analysis</u>. Please indicate if a) evidence synthesis was used in clinical trials analysis in which you have been involved over the last 10 years, to inform the following aspects and b) you think it should be used.**

	a) This was used			b) This should be used			I don't understand
	Yes	No	N/A*	Yes	No	Don't know	
External information about the treatment effect (including a meta-analysis)	☐	☐	☐	☐	☐	☐	☐
External information related to potential biases arising from trial conduct (e.g. blinding infeasible)	☐	☐	☐	☐	☐	☐	☐
External information about other quantities involved in the analysis (e.g. correlations or baseline event rates)	☐	☐	☐	☐	☐	☐	☐

* *N/A=not applicable (as I have not been a part of trial analysis)*

10. **Any other comments?**

Thank you for taking the time to complete this questionnaire.

Please hand this in to a member of the **INVEST team** during the conference, complete it online at bit.ly/investsurvey, or post
